# Translation and Validation of the Greek Food Allergy Quality of Life Questionnaire—Parental Form

**DOI:** 10.3390/pediatric16040090

**Published:** 2024-11-23

**Authors:** Emilia Vassilopoulou, Anna Comotti, Dafni Athanasaki, Gregorio Paolo Milani, Carlo Agostoni, George N. Konstantinou

**Affiliations:** 1 Department of Nutritional Sciences and Dietetics, International Hellenic University, 570 01 Thessaloniki, Greece; daphnieath1999@gmail.com; 2 Pediatric Unit, Fondazione IRCCS Ca’ Granda Ospedale Maggiore Policlinico, 20122 Milan, Italy; gregorio.milani@unimi.it (G.P.M.); carlo.agostoni@unimi.it (C.A.); 3 Occupational Health Unit, Foundation IRCCS Ca’ Granda Ospedale Maggiore Policlinico, 20122 Milan, Italy; anna.comotti@policlinico.mi.it; 4 Department of Clinical Sciences and Community Health, Università Degli Studi di Milano, 20122 Milan, Italy; 5 Department of Allergy and Clinical Immunology, 424 General Military Training Hospital, 564 29 Thessaloniki, Greece

**Keywords:** food allergy, children, quality of life, questionnaire, Greek

## Abstract

**Background:** The prevalence of food allergy is increasing, posing a significant health concern. Assessing health-related quality of life (HRQOL) in individuals with food allergies is crucial, and various questionnaires exist for this purpose. However, translation and validation of these tools are necessary to ensure cultural relevance. This study aimed to translate the FAQLQ-PF into Greek and perform a cross-sectional validation to assess its effectiveness in evaluating HRQOL among Greek children with food allergies. **Methods:** Parents of children aged 0 to 12 years diagnosed with food allergy completed the Greek version of the FAQLQ-PF, consisting of 30 items across three subscales. Socio-demographic and clinical data were collected. Statistical analyses included nonparametric tests, correlation analysis for validity, and Cronbach’s alpha for internal consistency. **Results:** Out of 85 participants, 81 were included. The FAQLQ-PF demonstrated excellent internal consistency (Cronbach’s α = 0.94). Validity evaluation indicated its ability to measure HRQOL in younger children. HRQOL in the Greek pediatric population was significantly correlated with the number of food allergies, symptoms, parental and child concerns, anxiety levels, and activity restrictions. However, sex and general health status were not significantly correlated with HRQOL. **Conclusions:** The Greek translation and validation of FAQLQ-PF provides insights into HRQOL among Greek children with food allergies. Factors such as anaphylactic reactions, epinephrine autoinjector usage, number of food allergies, and symptoms influenced HRQOL in this population.

## 1. Introduction

Food allergy (FA) represents a significant global public health concern affecting millions worldwide, particularly children, with prevalence rates reaching 10% among children and adolescents, though with considerable regional variation [1]. Its prevalence is notably higher among younger age groups than in older children [2,3,4,5]. This condition affects various aspects of an individual’s life, influencing daily routines and quality of life [2,3,4,5,6].

There is evidence suggesting a global increase in FA prevalence in recent decades, predominantly in Western-lifestyle countries [2,3,4,7,8]. However, the data supporting this trend are inconsistent [4,5] and prevalence rates uncertain [3].

FA management typically involves strict avoidance of the culprit foods and treatment of acute allergic reactions with rescue medication [4,9,10,11,12]. Children with food allergies and their families thus need to maintain continuous vigilance regarding what they are consuming in various situations and settings [9,11], significantly disrupting daily life due to the constant need to avoid the foods involved and the fear of accidental exposure [2,9,11,13,14]. Such a lifestyle, compounded by limited options for etiological treatment, adversely impacts health-related quality of life (HRQL), spanning physical, social, emotional, and psychosocial domains for both child and caregiver [2,4,9,11,13,14,15,16].

The negative impact of FA can also manifest as anxiety and/or restriction of daily activities [4,11]. This impact extends beyond the affected individual to burden caregivers, who manage diet and allergen avoidance, adding stress and responsibility [4,10,15,17,18,19,20]. Santos and colleagues underscored caregivers’ strong sense of fear, as they bear the responsibility for their charges and may not always be in a situation to observe and control what they eat and how they may react [4]. Therefore, it is essential to utilize valid and reliable instruments for assessing HRQL [12,20].

The Food Allergy Quality of Life Questionnaire—Parent Form (FAQLQ-PF) is among the most widely used food allergy–specific instruments for assessing the psychosocial impacts of the disease [2,12,20,21]. Validated in Europe as part of the EuroPrevall Project [22], the FAQLQ-PF measures the parental burden of managing a child with FA. It not only addresses the limitations in children’s lives caused by food safety concerns but also delves into the negative emotions evoked by these restrictions [20]. Additionally, the FAQLQ-PF includes items related to parental concern for their child’s physical and emotional health, parental and family stress, and the impact on family activities due to food allergy [20].

Recognizing the importance of cultural and socioeconomic context in health assessments, existing questionnaires need to be translated in a manner that aligns with the specific country’s conditions of use [22]. Therefore, this study aimed to translate the disease-specific HRQL questionnaire for children aged 0–12 years with food allergy—the FAQLQ-PF—into Greek and to perform a cross-sectional validation.

## 2. Materials and Methods

### 2.1. Participants and Procedure

A voluntary cross-sectional survey was conducted from January 2019 to October 2022 involving 81 children aged 0 to 12 years who had been diagnosed with food allergy by an allergist, along with their parents. Participants were recruited from the Department of Allergy and Clinical Immunology of the 424 General Military Training Hospital. Exclusion criteria included parents’ refusal to participate (*n* = 2), inability to communicate or complete the questionnaire (*n* = 3), presence of a serious concurrent illness that could affect HRQOL (*n* = 1), and extensive missing data (*n* = 4). A member of the research team conducted personal interviews with each participant to collect self-reported information. The questionnaire covered demographic and clinical data, including parents’ and child’s sex, types and number of foods avoided, types and number of symptoms experienced, episodes of anaphylactic reaction and use of epinephrine autoinjectors, concerns over food safety (rated on a 6-point scale from 0 = extremely unlikely to 6 = extremely likely), overall health of parents and children (rated from 0 = very poor to 6 = excellent), and parental concerns (rated from 0 = not at all to 5 = a lot). Subsequently, participants were asked to complete the FAQLQ-PF questionnaire, which had been translated into Greek and back-translated into English following World Health Organization (WHO) guidelines [23].

The full template of the questionnaire in the original language and English translation is available in Supplementary Materials.

The study was approved by the Committee for Research Ethics at Aristotle University of Thessaloniki (approval 1.272/20 October 2020) and was conducted in accordance with the World Medical Association’s Declaration of Helsinki ethical guidelines. Comprehensive information about the study’s objectives and procedures was provided to all participants, who provided written informed consent.

### 2.2. FAQLQ-PF Questionnaire

The FAQLQ-PF comprises 30 items divided into three sections: section A (applicable to all age groups, 13 items), section B (for children aged 4 to 12, 13 items), and section C (for children aged 7 to 12 years, 4 items). Items are rated on a 7-point Likert scale, ranging from 0 (no impact on the child’s quality of life) to 6 (extreme impact on the child’s quality of life). The questionnaire consists of three subscales assessing general emotional impact (items 2, 6, 7, 9, 10, 11, 23, 24, 25, and 26), food anxiety (items 1, 4, 5, 16, 17, 20, 21, and 29), and social and dietary limitations (items 3, 8, 12, 13, 14, 15, 18, 19, and 22). Subscale scores are calculated by averaging the scores of each subscale’s items. The total score is derived as the mean of the three subscales, with higher scores indicating a greater impact on quality of life [20].

The questionnaire comprises demographic and clinical data, including the sex of both the parents and child, the types and quantity of foods avoided, symptoms experienced, anaphylactic reaction incidents, and use of epinephrine autoinjectors. It also evaluates food safety concerns with a 6-point scale ranging from 0 (extremely unlikely) to 6 (extremely likely). Additionally, participants rate the general health of both the parents and child on a scale from 0 (very poor) to 6 (excellent) and express the parental concern level on a scale from 0 (none at all) to 5 (A lot). The translation of the FAQLQ-PF into Greek was conducted in accordance with WHO guidelines.

### 2.3. Statistical Analyses

The internal consistency of the scales and subscales was evaluated using Cronbach’s alpha. A Cronbach’s alpha value exceeding 0.8 indicates good internal consistency for the questionnaire [22].

To assess the validity of the questionnaire, we hypothesized that total FAQLQ-PF scores would correlate with variables theoretically expected to be associated with health-related quality of life in children with food allergies. These variables included the number of foods to which the child is allergic, the total number of allergic symptoms reported, levels of parental and child anxieties and concerns, stress levels, and the extent of activity restrictions for both the child and the family. We calculated Pearson correlation coefficients between questionnaire scores and these variables, with strong correlation thereby supporting convergent validity.

Second, we conducted a known-group validity analysis. We hypothesized that children with more severe food allergy experiences—such as a higher number of symptoms (>5) and allergens (>2), a history of anaphylaxis, or the use of epinephrine autoinjectors—would have higher FAQLQ-PF scores, indicating a greater impact on their quality of life. The Wilcoxon test was used for known-group validity.

To evaluate the adjusted effects of key variables on FAQLQ-PF scores, a multiple linear regression analysis was performed. This model considered the dichotomous variables of allergic symptoms (>5 or ≤5), number of food allergies (>2 or ≤2), history of anaphylaxis, and use of epinephrine autoinjectors. Parental and child worries about food safety were included as summary variables to represent the broader spectrum of concerns. The model was adjusted for the child’s sex, age, and overall health status.

A *p*-value < 0.05 was considered statistically significant. All analyses were conducted using R software (version 4.3.2).

## 3. Results

### 3.1. Descriptive Characteristics

**Figure 1:**
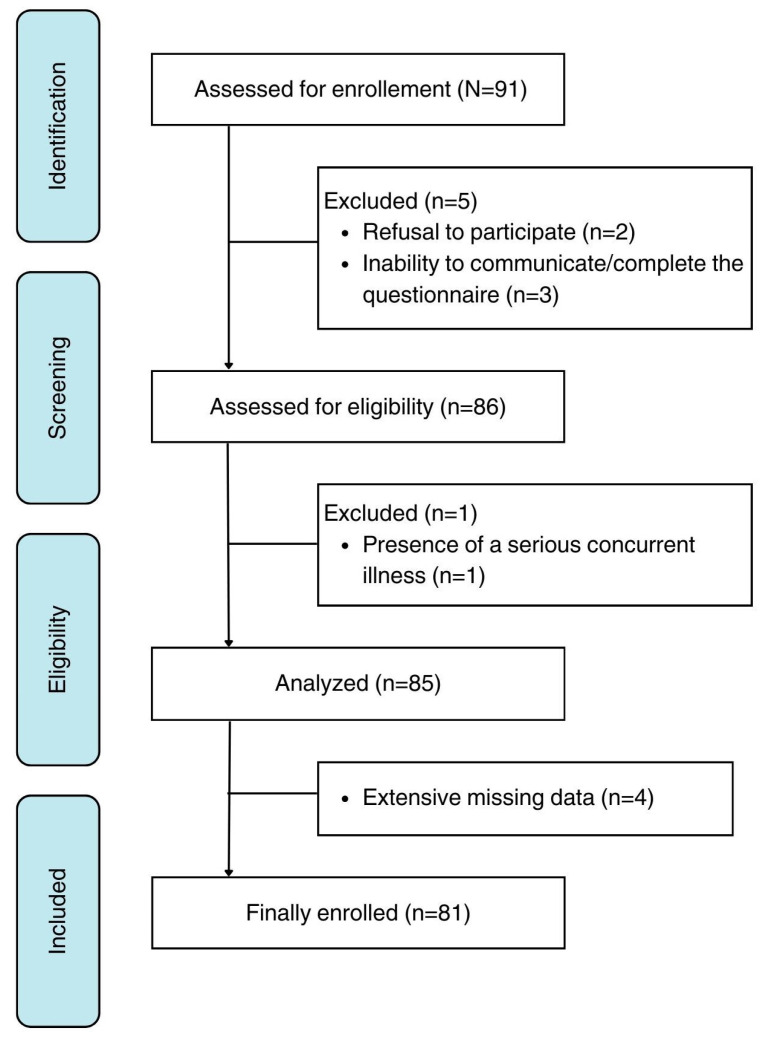
Study flowchart.

The questionnaire was fully completed by 81 parents or guardians, resulting in a response rate of 93% (Figure 1).

Descriptive characteristics of the children included in the cross-sectional validation are presented in Table 1. The sample had a majority of boys (68%) and a mean age of 4.7 years (±3.1). The largest age group was 0–3 years (43%), followed by 4–6 years (35%) and 7–12 years (22%). Among the children, 58% had experienced an anaphylactic reaction and 20% used epinephrine autoinjectors. The most common food allergies were to eggs (42%), nuts (36%), milk (35%), and peanuts (33%).

### 3.2. Reliability and Internal Consistency

In our study, the questionnaire demonstrated good internal consistency, with Cronbach’s alpha of 0.94 (95% confidence interval, 0.85–0.97). Subscale reliability was also high, with coefficients exceeding 0.85 (Table 2).

### 3.3. Validity

The FAQLQ-PF total scores were positively and significantly correlated with regard to parental and child anxieties and concerns, the parents’, partner’s, and family’s level of stress, and the extent of restrictions on family and child activities, indicating that high concerns were associated with a higher impact of food allergy on children’s quality of life. No significant correlation was found between FAQLQ-PF total scores and the overall health status of parents and children. Descriptive statistics of these variables and their correlation coefficients with FAQLQ-PF total scores are presented in Table 3.

Statistically significant differences were observed in our sample when stratified into three age groups, indicating an age-related increase in the impact of food allergies on quality of life (*p* = 0.02). The questionnaire also effectively differentiated between children who had experienced an anaphylactic reaction and those who had not, with parents of the former group reporting significantly higher scores (*p* < 0.01). Additionally, the impact of food allergies on quality of life was associated with the use of epinephrine autoinjectors, where use was linked to a higher impact (*p* < 0.01). Furthermore, the questionnaire distinguished differences based on the number of allergic symptoms, with children experiencing more than five symptoms showing higher scores compared to those with five or fewer symptoms (*p* < 0.001). Similarly, children allergic to more than two foods had significantly higher scores than those allergic to two or fewer foods (*p* = 0.03), highlighting the greater burden on quality of life with increased allergy severity (Table 4).

The adjusted multiple linear analysis showed that all the variables considered were associated with higher FAQLQ-PF scores, indicating a greater impact on quality of life. However, after adjusting for all other factors, statistical significance was observed only for children’s worries about food safety, which emerged as a significant risk factor for higher scores (Table 5).

## 4. Discussion

Assessment of HRQOL is essential for evaluating the impact of a disease on an individuals’ well-being. In the context of food allergies, HRQOL holds distinct importance due to the unique challenges and disruptions FA imposes on patients’ lives [22,24,25].

The efficacy of questionnaires designed to capture baseline data for pediatric patients with FA in Greece can be affected by cultural, culinary, and socioeconomic differences. Therefore, our objective was to develop a culturally tailored Greek version of the FAQLQ-PF and to validate its effectiveness in evaluating HRQOL among Greek pediatric patients.

Our statistical analysis revealed strong reliability and internal consistency across the FAQLQ-PF and its specific domains, including emotional impact, food anxiety, and social and dietary limitations. Consequently, the FAQLQ-PF is confirmed to be an excellent tool for HRQOL assessment within the Greek pediatric population, demonstrating its effective applicability for children aged 0–12 years.

Finally, validity assessment through correlation of the total FAQLQ-PF score with the collected variables revealed significant associations with parental and children’s anxieties, parental concerns and anxiety regarding their child, activity limitations, and the number of food allergies and symptoms experienced by the child. Conversely, no association was found between the FAQLQ-PF scores and the general health status of parents and children. Previous research by Dunn and colleagues highlighted an increased risk of negative perceptions of HRQL associated with poor maternal general health status [12]. This finding may suggest and reinforce the notion that the FAQLQ-PF, being disease-specific, might not adequately capture broader health variables, such as overall health status.

The cross-sectional validation of the Greek version of the FAQLQ-PF provides valuable insights into HRQOL in the study population. Notably, no differences in HRQOL were observed when comparing sexes, aligning with findings from Manso and colleagues [22]. This finding contrasts with other studies indicating sex-specific disparities in FAQLQ-PF scores, where females often report a reduced quality of life [26,27]. In terms of age, older individuals tend to experience a greater impact on their quality of life due to FA. These findings align with previous studies indicating varying HRQOL outcomes across age groups, with the 0- to 3-year [22], >5-year [27] and >9-year [12] groups reporting relatively better HRQOL compared to older patients.

This suggests that with increasing age, FA patients develop a heightened awareness of the severity of symptoms [9], ranging from mild to life-threatening [1]. Consequently, these patients may perceive their quality of life as being more adversely affected compared to those experiencing less severe food allergy reactions [9].

The impact of anaphylaxis on quality of life is highlighted by findings that suggest a more pronounced effect when epinephrine autoinjectors are utilized, indicating a significant influence on quality-of-life assessments [9]. This finding contrasts with previous studies [11,22,26] that reported that experiencing anaphylaxis did not contribute to impairment in HRQL. Nonetheless, the use of epinephrine autoinjectors appeared to have an impact on the overall FAQOL-PF score.

Previous studies have revealed that both prescription and prior utilization of an epinephrine autoinjector [26] were associated with diminished HRQL impairment [28]. In line with previous studies [9,11,12] and contrary to Manso and colleagues [22], our data suggest that the number of foods implicated affects HRQOL and total FAQLQ-PF score. Specifically, a greater number of implicated foods is associated with more pronounced effects on quality of life.

In this study, the FAQLQ-PF was successfully translated into Greek and validated for use. One notable strength was the exclusive use of participants from the Department of Allergy who were well characterized according to FA guidelines as food-allergic. Additionally, the study also benefited from a well-distributed age range among participants, ensuring representation across all age groups. Moreover, the translation process from English to Greek was meticulously conducted to ensure cultural adaptation.

### Limitations

The study has several limitations. Although data collection was conducted through structured interviews by a trained team member to enhance consistency and reduce misinterpretation, the reliance on self-reported information introduces potential biases and subjectivity. To address this, we ensured that key variables, such as the diagnosis of anaphylaxis, were corroborated by medical documentation wherever possible.

Additionally, the sampling was confined to a single region of Greece, which may limit the generalizability of findings. However, the uniformity of the Greek language and healthcare practices likely mitigates this concern. While the sample size was adequate for initial validation, it was relatively small and may not fully represent the diverse experiences of children with varying allergy severity or socioeconomic backgrounds. To address this, we recruited participants across a wide age range and collected comprehensive clinical and demographic data to ensure a representative dataset.

Finally, the study did not include a formal construct validity assessment using other validated HRQoL tools, such as the FAIM or SF-8 score. Instead, we supported validity by analyzing correlations with theoretically relevant variables and conducting known-group validity testing. While these approaches strengthen the FAQLQ-PF’s validity, future studies should aim to include direct comparisons with other validated instruments. Additionally, the cross-sectional design limits the ability to assess changes over time or the impact of interventions, which should be explored in future research.

## 5. Conclusions

The translation and validation of the FAQLQ-PF into Greek represent a significant step forward in understanding and managing the impact of food allergies on children and their families within a cultural context. This study confirms the instrument’s reliability and validity in capturing the multifaceted psychosocial effects of food allergies, providing an insight into the challenges faced by affected families. The strong correlations between FAQLQ-PF scores and key variables related to food allergy severity and psychosocial stress underline the questionnaire’s efficacy in assessing HRQOL. Furthermore, the lack of correlation between overall health status and FAQLQ-PF scores suggests the instrument’s focused applicability in addressing food allergy–specific concerns rather than general health. By enabling precise measurement of the quality-of-life impacts associated with food allergies, the Greek FAQLQ-PF facilitates targeted interventions and support that can significantly improve the well-being of children and their caregivers, emphasizing the critical role of culturally and linguistically tailored healthcare tools in global health contexts.

## Figures and Tables

**Table 1:** Descriptive characteristics of the sample involved in the cross-sectional validation.

Variable	Frequency (%) (*n* = 81)
Sex	
Boy	55 (68%)
Girl	26 (32%)
Age groups	
0–3	35 (43%)
4–6	28 (35%)
7–12	18 (22%)
Age (years)	4.7 (±3.1) ^1^
Sex of parent completing the questionnaire	
Female	67 (83%)
Male	14 (17%)
Epinephrine autoinjector usage ^2^	16 (20%)
Anaphylactic reaction	47 (58%)
Food allergy	
Eggs	34 (42%)
Nuts	29 (36%)
Milk	28 (35%)
Peanuts	27 (33%)
Fish	18 (22%)
Seeds	12 (15%)
Wheat	12 (15%)
Lentils	6 (7%)
Fruits	6 (7%)
Peas	4 (5%)
Shellfish	4 (5%)
Soya	4 (5%)
Legumes	3 (4%)
Chicks	3 (4%)
Chicken	3 (4%)
Vegetables	3 (4%)
Moisture/Mold/Wool	2 (2%)
Spices	2 (2%)
Walnuts	1 (1%)

^1^ Mean (±SD); ^2^ epinephrine autoinjectors used during allergic reactions, not just prescribed or made available.

**Table 2:** Reliability and internal consistency.

	Cronbach’s Alpha
Emotional impact	0.91 (0.79, 0.96)
Food anxiety	0.86 (0.58, 0.96)
Social and dietary limitations	0.89 (0.83, 0.93)
FAQLQ-PF ^1^	0.94 (0.85, 0.97)

^1^ FAQLQ-PF = Food Allergy Quality of Life Questionnaire—Parent Form.

**Table 3:** Associations between Food Allergy Quality of Life Questionnaire—Parent Form (FAQLQ-PF) total scores and parental concerns and the general health status of parents and children.

	NAs	Mean (SD ^1^)	Median (IQR ^2^)	Range	Correlation with FAQLQ-PF ^3^
Parents’ worries	6	3.34 (1.12)	3.5 (2.5–4.25)	0–5	0.47 ***
Children’s worries	16	2.20 (1.46)	2.25 (1, 3.25)	0–5	0.58 ***
Parents’ general health	2	4.71 (0.89)	5 (4–5)	2–6	−0.13
Child’s general health	2	5.01 (0.72)	5 (5–5)	2–6	−0.21
Concerns about child’s physical health	2	2.63 (1.36)	3 (1–4)	0–4	0.41 ***
Concerns about child’s emotional well-being	2	2.81 (1.34)	3 (1–4)	0–4	0.47 ***
Parents’ level of stress caused by food allergy	2	3.14 (1.14)	4 (1–4)	0–4	0.55 ***
Partner’s level of stress caused by food allergy	3	2.96 (1.22)	3 (2–4)	0–4	0.42 ***
Family’s level of stress caused by food allergy	2	2.87 (1.15)	3 (2–4)	0–4	0.53 ***
Limited family activities	2	1.44 (1.27)	2 (0–2)	0–4	0.52 ***
Limited child activities	2	1.43 (1.16)	1 (0–2)	0–4	0.51 ***

^1^ SD = standard deviation; ^2^ IQR = interquartile range; ^3^ FAQLQ-PF = Food Allergy Quality of Life Questionnaire—Parent Form. *** *p* < 0.001 considered statistically significant.

**Table 4:** Association between Food Allergy Quality of Life Questionnaire—Parent Form (FAQLQ-PF) scores and dichotomous variables.

Variable	Valid Count N (%)	FAQLQ-PF ^1^ Mean (SD ^2^)	FAQLQ-PF ^1^ Median (IQR ^3^)
Sex			
Boy	55 (68)	1.93 (1.29)	1.83 (0.7–2.6)
Girl	26 (32)	2.15 (1.28)	2.23 (1.0–3.2)
Anaphylactic reaction *			
No	24 (42)	1.56 (1.21)	1.62 (0.5–2.4)
Yes	47 (58)	2.32 (1.25)	2.31 (1.3–3.1)
Epinephrine autoinjector usage **			
No	64 (79)	1.80 (1.27)	1.71 (0.8–2.6)
Yes	17 (21)	2.73 (1.08)	2.78 (1.9–3.6)
Number of symptoms ***			
>5	35 (43)	2.50 (1.27)	2.55 (1.83–3.11)
≤5	46 (57)	1.61 (1.13)	1.48 (0.53–2.40)
Number of foods child is allergic to *			
>2	52 (64)	2.43 (1.14)	2.31 (1.78–2.89)
≤2	29 (36)	1.76 (1.31)	1.63 (0.58–2.73)

^1^ FAQLQ-PF = Food Allergy Quality of Life Questionnaire—Parent Form; ^2^ SD = standard deviation; ^3^ IQR = interquartile range; * *p* < 0.05; ** *p* < 0.01; *** *p* < 0.001 (Wilcoxon test) considered statistically significant.

**Table 5:** Multiple linear regression model.

	β (*p*-Value)
Anaphylactic reaction	0.17 (*p* = 0.57)
Epinephrine autoinjector usage	0.20 (*p* = 0.54)
Parents’ worries	0.26 (*p* = 0.58)
Child’s worries	0.31 (*p* = 0.007)
Number of symptoms > 5	0.24 (*p* = 0.42)
Number of food allergies > 2	0.11 (*p* = 0.68)

Each coefficient adjusted by the other variables and by age, sex, and health status.

## Data Availability

Data are available upon request to the corresponding author.

## Supplementary Materials

The following supporting information can be downloaded at: https://www.mdpi.com/article/10.3390/pediatric16040090/s1.
