# Supplementary material for: Translation and Validation of the Greek Food Allergy Quality of Life Questionnaire—Parental Form
Source: Pediatr Rep. 2024 Nov 23;16(4):1054–63. doi: 10.3390/pediatric16040090 (PMC11587411; doi:10.3390/pediatric16040090)
Supplement: Supplementary file 1 [file pediatrrep-16-00090-s001.zip › FAQLQ_PF_GR_EN_TRANSLATION.docx]

ΕΡΩΤΗΜΑΤΟΛΟΓΙΟ ΠΟΙΟΤΗΤΑΣ ΖΩΗΣ ΑΣΘΕΝΩΝ ΜΕ ΤΡΟΦΙΚΗ ΑΛΛΕΡΓΙΑ

- ΕΝΤΥΠΟ ΓΟΝΕΩΝ (0-12 ΕΤΩΝ)

ΕΡΩΤΗΜΑΤΟΛΟΓΙΟ ΠΟΙΟΤΗΤΑΣ ΖΩΗΣ ΑΣΘΕΝΩΝ ΜΕ ΤΡΟΦΙΚΗ ΑΛΛΕΡΓΙΑ – ΕΝΤΥΠΟ ΓΟΝΕΩΝ

ΓΙΑ ΠΑΙΔΙΑ ΗΛΙΚΙΑΣ 0-12 ΧΡΟΝΩΝ

Οδηγίες προς τους γονείς:

| *Τα ακόλουθα είναι σενάρια που οι γονείς μας έχουν πει ότι επηρεάζουν την ποιότητα ζωής των παιδιών τους εξαιτίας της τροφικής αλλεργίας.*  *Παρακαλούμε υποδείξτε μας πόσο αντίκτυπο έχει κάθε σενάριο στην ποιότητα ζωής του παιδιού σας βάζοντας ένα* ***τικ*** *ή ένα* ***x*** *στο καθένα από τα αριθμημένα κουτιά 0-6.*  *Όλες οι πληροφορίες που δίνονται είναι εντελώς εμπιστευτικές. Αυτό το ερωτηματολόγιο θα αναγνωριστεί μόνο από έναν κωδικό αριθμό.* | Επιλογές απάντησης  **0 = καθόλου**  **1 = λιγάκι**  **2 = ελαφρώς**  **3 = μέτρια**  **4 = αρκετά**  **5 = πάρα πολύ**  **6 = υπερβολικά** |
| --- | --- |

Υπάρχουν 4 ενότητες σ’ αυτό το ερωτηματολόγιο: Α, Β, Γ και Δ.

- Εάν το παιδί σας είναι ηλικίας 0 με 3 ετών, παρακαλούμε απαντήστε την ενότητα Α και Δ έως ΣΤ.
- Εάν το παιδί σας είναι ηλικίας 4 με 6 ετών, παρακαλούμε απαντήστε την ενότητα Α και Β και Δ έως ΣΤ.
- Εάν το παιδί σας είναι ηλικίας 7 ετών και πάνω παρακαλώ απαντήστε σε όλες τις ενότητες.

| **Καθόλου 🡪 Υπερβολικά** | | | | | | | | |
| --- | --- | --- | --- | --- | --- | --- | --- | --- |
| **Εξαιτίας της τροφικής αλλεργίας το παιδί**  **μου νιώθει…** | | **0** | **1** | **2** | **3** | **4** | **5** | **6** |
| 1 | ανήσυχο σχετικά με το φαγητό |  |  |  |  |  |  |  |
| 2 | διαφορετικό από τα άλλα παιδιά |  |  |  |  |  |  |  |
| 3 | αγχωμένο από τους διατροφικούς περιορισμούς |  |  |  |  |  |  |  |
| 4 | φοβάται να δοκιμάσει άγνωστα φαγητά |  |  |  |  |  |  |  |
| 5 | ανησυχεί που ανησυχώ ότι αυτός/αυτή  θα έχει αντίδραση στο φαγητό |  |  |  |  |  |  |  |

**ΕΝΟΤΗΤΑ Α: ΓΙΑ ΟΛΕΣ ΤΙΣ ΗΛΙΚΙΑΚΕΣ ΟΜΑΔΕΣ**

| **Καθόλου 🡪 Υπερβολικά** | | | | | | | | |
| --- | --- | --- | --- | --- | --- | --- | --- | --- |
| **Εξαιτίας της τροφικής αλλεργίας το παιδί**  **μου…** | | **0** | **1** | **2** | **3** | **4** | **5** | **6** |
| 6 | βιώνει σωματική δυσφορία |  |  |  |  |  |  |  |
| 7 | βιώνει συναισθηματική δυσφορία |  |  |  |  |  |  |  |
| 8 | έχει έλλειψη ποικιλίας στη διατροφή  του |  |  |  |  |  |  |  |

| **Καθόλου 🡪 Υπερβολικά** | | | | | | | | |
| --- | --- | --- | --- | --- | --- | --- | --- | --- |
| **Εξαιτίας της τροφικής αλλεργίας το παιδί μου έχει επηρεαστεί αρνητικά από…** | | **0** | **1** | **2** | **3** | **4** | **5** | **6** |
| 9 | το γ το γεγονός ότι του αφιερώνεται  π περισσότερη προσοχή από άλλα παιδιά  πα της ηλικίας του |  |  |  |  |  |  |  |
| 10 | το γεγονός ότι πρέπει να μεγαλώσει πιο  γρήγορα από άλλα παιδιά της ηλικίας  του |  |  |  |  |  |  |  |
| 11 | το γεγονός ότι το περιβάλλον του  γίνεται πιο περιορισμένο από των  άλλων παιδιών |  |  |  |  |  |  |  |

| **Καθόλου 🡪 Υπερβολικά** | | | | | | | | |
| --- | --- | --- | --- | --- | --- | --- | --- | --- |
| **Εξαιτίας της τροφικής αλλεργίας το**  **κοινωνικό περιβάλλον του παιδιού μου είναι περιορισμένο εξαιτίας των περιορισμών σε…** | | **0** | **1** | **2** | **3** | **4** | **5** | **6** |
| 12 | εστιατόρια που μπορούμε να πάμε  με ασφάλεια ως οικογένεια |  |  |  |  |  |  |  |
| 13 | προορισμούς διακοπών που μπορούμε να  πάμε με ασφάλεια ως οικογένεια |  |  |  |  |  |  |  |

**ΕΝΟΤΗΤΑ Β: ΓΙΑ ΠΑΙΔΙΑ ΗΛΙΚΙΑΣ 4 ΜΕ 12 ΕΤΩΝ**

| **Καθόλου 🡪 Υπερβολικά** | | | | | | | | |
| --- | --- | --- | --- | --- | --- | --- | --- | --- |
| **Εξαιτίας της τροφικής αλλεργίας έχει  περιοριστεί η ικανότητα του παιδιού μου  να συμμετέχει…** | | **0** | **1** | **2** | **3** | **4** | **5** | **6** |
| 14 | σε κοινωνικές δραστηριότητες σε  σπίτια άλλων ανθρώπων (ολονύκτια  διαμονή σε σπίτια τρίτων, πάρτι,  παιχνίδι) |  |  |  |  |  |  |  |
| 15 | σε προσχολικές/σχολικές εκδηλώσεις  που συμπεριλαμβάνουν φαγητό(πάρτι  στη τάξη/κεράσματα/μεσημεριανό) |  |  |  |  |  |  |  |

| **Καθόλου 🡪 Υπερβολικά** | | | | | | | | |
| --- | --- | --- | --- | --- | --- | --- | --- | --- |
| **Εξαιτίας της τροφικής αλλεργίας, το παιδί**  **μου αισθάνεται…** | | **0** | **1** | **2** | **3** | **4** | **5** | **6** |
| 16 | ανήσυχο όταν πηγαίνει σε άγνωστα μέρη |  |  |  |  |  |  |  |
| 17 | την ανησυχία ότι πρέπει πάντα να είναι  προσεκτικό σχετικά με τα τρόφιμα |  |  |  |  |  |  |  |
| 18 | ότι δε μπορεί να συμμετέχει σε  δραστηριότητες που συμπεριλαμβάνουν  φαγητό |  |  |  |  |  |  |  |
| 19 | αναστατωμένο ότι οι οικογενειακές  κοινωνικές έξοδοι έχουν περιοριστεί από  την ανάγκη να προγραμματιστούν εκ των  προτέρων |  |  |  |  |  |  |  |
| 20 | ανήσυχο για τυχαία κατανάλωση ενός  συστατικού στο οποίο είναι αλλεργικό |  |  |  |  |  |  |  |
| 21 | ανήσυχο όταν τρώει με άγνωστους  ενήλικες/παιδιά |  |  |  |  |  |  |  |
| 22 | αγχωμένο από τους κοινωνικούς  περιορισμούς |  |  |  |  |  |  |  |

| **Καθόλου 🡪 Υπερβολικά** | | | | | | | | |
| --- | --- | --- | --- | --- | --- | --- | --- | --- |
| **Εξαιτίας της τροφικής αλλεργίας το παιδί…** | | **0** | **1** | **2** | **3** | **4** | **5** | **6** |
| 23 | είναι γενικά περισσότερο ανήσυχο από άλλα παιδιά της ηλικίας του |  |  |  |  |  |  |  |
| 24 | είναι γενικά περισσότερο προσεκτικό  από άλλα παιδιά της ηλικίας του |  |  |  |  |  |  |  |
| 25 | δεν έχει τόση αυτοπεποίθηση όσο άλλα  παιδιά της ηλικίας του σε κοινωνικές  καταστάσεις |  |  |  |  |  |  |  |
| 26 | εύχεται η τροφική του αλλεργία να  εξαφανιστεί |  |  |  |  |  |  |  |

**ΕΝΟΤΗΤΑ Γ: ΓΙΑ ΠΑΙΔΙΑ ΗΛΙΚΙΑΣ 7 ΜΕ 12 ΕΤΩΝ**

| **Καθόλου 🡪 Υπερβολικά** | | | | | | | | |
| --- | --- | --- | --- | --- | --- | --- | --- | --- |
| **Εξαιτίας της τροφικής αλλεργίας , το παιδί  μου νιώθει…** | | **0** | **1** | **2** | **3** | **4** | **5** | **6** |
| 27 | ανήσυχο σχετικά με το μέλλον του/της (ευκαιρίες, σχέσεις) |  |  |  |  |  |  |  |
| 28 | ότι πολλοί άνθρωποι δεν καταλαβαίνουν  τη σοβαρή φύση των τροφικών αλλεργιών  του |  |  |  |  |  |  |  |
| 29 | ανήσυχο για την ανεπαρκή σήμανση των προϊόντων διατροφής |  |  |  |  |  |  |  |
| 30 | ότι η τροφική αλλεργία περιορίζει την ζωή του γενικότερα |  |  |  |  |  |  |  |

Σας ευχαριστούμε που συμπληρώσατε αυτό το ερωτηματολόγιο. Θα ήμασταν ευγνώμονες εάν απαντούσατε τώρα σε μερικές ερωτήσεις σχετικά με την τροφική αλλεργία του παιδιού σας.

**ΕΝΟΤΗΤΑ Δ: ΓΙΑ ΟΛΕΣ ΤΙΣ ΗΛΙΚΙΑΚΕΣ ΟΜΑΔΕΣ**

**ΜΕΡΟΣ 1^Ο^**
Η τροφική αλλεργία του παιδιού μου:

**Ε1**. Τι φύλο είστε; (ο γονέας που συμπληρώνει το ερωτηματολόγιο)

| ΑΝΤΡΑΣ |  | ΓΥΝΑΙΚΑ |  |
| --- | --- | --- | --- |

**Ε2**. Τι φύλο είναι το παιδί σου;

| ΑΓΟΡΙ |  | ΚΟΡΙΤΣΙ |  |
| --- | --- | --- | --- |

**Ε3**. Τι ηλικία έχει το παιδί με τροφική αλλεργία; Έτη ____ Μήνες ____

**Ε4**. Σε τι είδους τροφή/ές είναι το παιδί σας αλλεργικό; Σημειώστε όπου χρειάζεται.

🞎 Φιστίκι 🞎 Ξηρούς καρπούς 🞎 Γάλα

🞎 Σιτάρι 🞎 Σόγια 🞎 Αυγό

🞎 Οστρακοειδή 🞎 Φρούτα 🞎 Ψάρι

🞎 Λαχανικά 🞎 Σουσάμι 🞎 Άλλο

Παρακαλώ προσδιορίστε το ‘άλλο’ :

**Ε5**. Μετά την κατανάλωση ποιας τροφής παρουσίασε το παιδί σας την πιο σοβαρή αντίδραση;

**Ε6**. Εμφάνισε το παιδί σας αναφυλακτική αντίδραση; Ναι 🞎 Όχι 🞎

**Ε7**. Αν ‘ναι’ πόσο πρόσφατη ήταν η αντίδραση; Σημειώστε με «Χ» όπου χρειάζεται.

🞎 Πολύ πρόσφατη

🞎 6 με 12 μήνες πριν

🞎 Περίπου 1 χρόνο πριν

🞎 Περίπου 2 χρόνια πριν

🞎 Περισσότερο από 2 χρόνια πριν

**Ε8**.α. Έχει χορηγηθεί ποτέ στο παιδί σας Anapen/ αδρεναλίνη/ επινεφρίνη;

Ναι 🞎 Όχι 🞎

**Ε8**.β. Η χορήγηση μιας Anapen/ αδρεναλίνης/ επινεφρίνης:

1. Καθησυχάζει Εσάς Το παιδί σας
2. Προκαλεί ανησυχία Σε εσάς Στο παιδί σας

**Ε9**. Ποιος διάγνωσε στο παιδί σας με τροφική αλλεργία;

| Οικογενειακός γιατρός |  |
| --- | --- |
| Αλλεργιολόγος |  |
| Παιδίατρος |  |
| Δερματολόγος |  |
| Διαιτολόγος |  |
| Άλλος ιατρός |  |

**Ε10**. Ποια συμπτώματα εμφανίζει το παιδί σας στα πλαίσια της τροφικής αλλεργίας; Σημειώστε με «Χ» όπου χρειάζεται.

|  | Φαγούρα στο στόμα |  |  | Συριγμό |
| --- | --- | --- | --- | --- |
|  | Φαγούρα στο λαιμό |  |  | Βήχα |
|  | Φαγούρα στα αυτιά |  |  | Φαγούρα στο δέρμα |
|  | Φαγούρα στα χείλια |  |  | Ερυθρότητα του δέρματος |
|  | Ρινική καταρροή |  |  | Αύξηση εκζέματος |
|  | Συμφόρηση (βουλωμένη μύτη) |  |  | Κνίδωση |
|  | Φτάρνισμα |  |  | Οίδημα του δέρματος |
|  | Φαγούρα ματιών |  |  | Ναυτία |
|  | Δάκρυα |  |  | Κοιλιακές κράμπες |
|  | Κόκκινα μάτια |  |  | Εμετός |
|  | Σφίξιμο στο λαιμό |  |  | Διάρροια |
|  | Δυσκολία στη κατάποση |  |  | Ζαλάδα |
|  | Βραχνάδα |  |  | Αίσθημα παλμών |
|  | Δυσκολία αναπνοής |  |  | Αδυναμία να σταθεί |
|  | Λαχάνιασμα |  |  | Απώλεια συνείδησης |

**Ε11**. Πόσο συχνά το παιδί σας συναντά άλλο παιδί με τροφική αλλεργία;

| Ποτέ |  |
| --- | --- |
| Σπάνια |  |
| Μερικές φορές |  |
| Συχνά |  |

**ΕΝΟΤΗΤΑ Ε: ΓΙΑ ΟΛΕΣ ΤΙΣ ΗΛΙΚΙΑΚΕΣ ΟΜΑΔΕΣ**

**ΜΕΡΟΣ 2^Ο^ :** Οι ανησυχίες, οι δικές σας και του παιδιού σας σχετικά με την ασφάλεια των τροφίμων.

| **0 = υπερβολικά απίθανο**  **1 = πολύ απίθανο**  **2 = κάπως απίθανο**  **3 = πιθανό**  **4 = αρκετά πιθανό**  **5 = πολύ πιθανό**  **6 = υπερβολικά πιθανό** |
| --- |

*Παρακαλώ απαντήστε τις ακόλουθες ερωτήσεις με βάση την κλίμακα των 6 βαθμών που υπάρχει στα δεξιά.*

**Ε1**. Τι πιθανότητα πιστεύετε **ΕΣΕΙΣ** ότι έχει το παιδί σας να…

| **ΕΡΩΤΗΣΗ** | | **ΚΛΙΜΑΚΑ ΤΩΝ 6 ΒΑΘΜΩΝ** | | | | | | |
| --- | --- | --- | --- | --- | --- | --- | --- | --- |
|  |  | 0 | 1 | 2 | 3 | 4 | 5 | 6 |
| 1 | καταναλώσει τυχαία τροφή στην οποία είναι αλλεργικό; |  |  |  |  |  |  |  |
| 2 | έχει μια σοβαρή αντίδραση εάν η τροφή ληφθεί τυχαία; |  |  |  |  |  |  |  |
| 3 | πεθάνει από την τροφική του αλλεργία μετά από την κατανάλωση στο μέλλον; |  |  |  |  |  |  |  |
| 4 | να αυτοθεραπευτεί ή να λάβει αποτελεσματική θεραπεία από άλλους (συμπεριλαμβανομένης της χορήγησης αδρεναλίνης) εάν λάβει τυχαία τροφή στην οποία είναι αλλεργικό; |  |  |  |  |  |  |  |

**Ε2**. Τι πιθανότητα θεωρεί το **ΙΔΙΟ** το παιδί ότι έχει να…..;

| **ΕΡΩΤΗΣΗ** | | **ΚΛΙΜΑΚΑ ΤΩΝ 6 ΒΑΘΜΩΝ** | | | | | | |
| --- | --- | --- | --- | --- | --- | --- | --- | --- |
|  |  | 0 | 1 | 2 | 3 | 4 | 5 | 6 |
| 1 | καταναλώσει τυχαία τροφή στην οποία είναι αλλεργικό; |  |  |  |  |  |  |  |
| 2 | έχει μια σοβαρή αντίδραση εάν η τροφή ληφθεί τυχαία; |  |  |  |  |  |  |  |
| 3 | πεθάνει από την τροφική του αλλεργία μετά από την κατανάλωση στο μέλλον; |  |  |  |  |  |  |  |
| 4 | να αυτοθεραπευτεί ή να λάβει αποτελεσματική θεραπεία από άλλους (συμπεριλαμβανομένης της χορήγησης αδρεναλίνης) εάν λάβει τυχαία τροφή στο οποίο είναι αλλεργικό; |  |  |  |  |  |  |  |

| **0-2** |  |
| --- | --- |
| **3-6** |  |
| **7-10** |  |
| **10+** |  |

**Ε3**. Πόσες τροφές πρέπει το παιδί σου να αποφεύγει;

**ΕΝΟΤΗΤΑ ΣΤ :ΓΙΑ ΟΛΕΣ ΤΙΣ ΗΛΙΚΙΑΚΕΣ ΟΜΑΔΕΣ**

**ΜΕΡΟΣ 3^Ο^ :** Οι ανησυχίες σας ως γονιός

**Ε1**. Πώς θα περιγράφατε…..

Α. Τη γενική σας υγεία; (τη **ΔΙΚΗ** σας)

| ΑΡΙΣΤΗ |  |
| --- | --- |
| ΠΟΛΥ ΚΑΛΗ |  |
| ΚΑΛΗ |  |
| ΜΕΤΡΙΑ |  |
| ΟΧΙ ΤΟΣΟ ΚΑΛΗ |  |
| ΚΑΚΗ |  |
| ΠΟΛΥ ΚΑΚΗ |  |

Β. Τη γενική υγεία του παιδιού σας;

| ΑΡΙΣΤΗ |  |
| --- | --- |
| ΠΟΛΥ ΚΑΛΗ |  |
| ΚΑΛΗ |  |
| ΜΕΤΡΙΑ |  |
| ΟΧΙ ΤΟΣΟ ΚΑΛΗ |  |
| ΚΑΚΗ |  |
| ΠΟΛΥ ΚΑΚΗ |  |

**Ε2**. Εξαιτίας της τροφικής αλλεργίας πόση ανησυχία σας προκαλεί το καθένα από τα παρακάτω;

Α. Η σωματική υγεία του παιδιού σας

| ΚΑΘΟΛΟΥ |  | ΛΙΓΑΚΙ |  | ΜΕΡΙΚΩΣ |  | ΑΡΚΕΤΑ |  | ΠΟΛΥ |  |
| --- | --- | --- | --- | --- | --- | --- | --- | --- | --- |

Β. Η καλή συναισθηματική κατάσταση του παιδιού σας

| ΚΑΘΟΛΟΥ |  | ΛΙΓΑΚΙ |  | ΜΕΡΙΚΩΣ |  | ΑΡΚΕΤΑ |  | ΠΟΛΥ |  |
| --- | --- | --- | --- | --- | --- | --- | --- | --- | --- |

**Ε3**. Πόσο στρες προκαλεί η τροφική αλλεργία του παιδιού σας…

Α. σε εσάς;

| ΚΑΘΟΛΟΥ |  | ΛΙΓΑΚΙ |  | ΜΕΡΙΚΩΣ |  | ΑΡΚΕΤΑ |  | ΠΟΛΥ |  |
| --- | --- | --- | --- | --- | --- | --- | --- | --- | --- |

Β. Στον σύντροφο/σύζυγο σας;

| ΚΑΘΟΛΟΥ |  | ΛΙΓΑΚΙ |  | ΜΕΡΙΚΩΣ |  | ΑΡΚΕΤΑ |  | ΠΟΛΥ |  |
| --- | --- | --- | --- | --- | --- | --- | --- | --- | --- |

Γ. Στην οικογένεια σας;

| ΚΑΘΟΛΟΥ |  | ΛΙΓΑΚΙ |  | ΜΕΡΙΚΩΣ |  | ΑΡΚΕΤΑ |  | ΠΟΛΥ |  |
| --- | --- | --- | --- | --- | --- | --- | --- | --- | --- |

**Ε4**. Έχει περιορίσει η τροφική αλλεργία τον τύπο δραστηριοτήτων…

Α. που μπορείτε να κάνετε ως οικογένεια;

| ΚΑΘΟΛΟΥ |  | ΛΙΓΑΚΙ |  | ΜΕΡΙΚΩΣ |  | ΑΡΚΕΤΑ |  | ΠΟΛΥ |  |
| --- | --- | --- | --- | --- | --- | --- | --- | --- | --- |

Β. που το παιδί σας μπορεί να συμμετέχει;

| ΚΑΘΟΛΟΥ |  | ΛΙΓΑΚΙ |  | ΜΕΡΙΚΩΣ |  | ΑΡΚΕΤΑ |  | ΠΟΛΥ |  |
| --- | --- | --- | --- | --- | --- | --- | --- | --- | --- |

Σας ευχαριστούμε για το χρόνο που αφιερώσατε για να συμπληρώσετε το παρόν ερωτηματολόγιο. Σας είμαστε ευγνώμων.

Quality of Life Questionnaire for Patients with Food Allergy

Parent Form (0-12 years)

Quality of Life Questionnaire for Patients with Food Allergy

Parent Form for children aged 0-12 years

Instructions to parents:

| *The following are scenarios which parents have said affect their children’s quality of life because of food allergy.*  *Please indicate how much impact each scenario has on your child’s quality of life by placing a tick or x in each of the boxes numbered 0-6.*  *All the information given is absolutely confidential. This questionnaire will only be identified by a code number.* | Response options:  **0 = not at all**  **1 = a little bit**  **2 = slightly**  **3 = moderately**  **4 = quite a bit**  **5 = very much**  **6 = extremely** |
| --- | --- |

**There are four sections in this questionnaire: A, B, C, and D.**

- **If your child is aged 0-3 years, please answer ONLY section A and from D to the end.**
- **If your child is aged 4-6 years, please answer sections A and B and from D to the end.**
- **If your child is aged 7 years and above, please answer all the sections.**

| **Not at all 🡪 Extremely** | | | | | | | | |
| --- | --- | --- | --- | --- | --- | --- | --- | --- |
| **Because of food allergy, my child feels...** | | **0** | **1** | **2** | **3** | **4** | **5** | **6** |
| 1 | Worried about food |  |  |  |  |  |  |  |
| 2 | Different from other children |  |  |  |  |  |  |  |
| 3 | Anxious because of dietary restrictions |  |  |  |  |  |  |  |
| 4 | Afraid to try unknown foods |  |  |  |  |  |  |  |
| 5 | Concerned that I am worried that he/she will have a reaction to food |  |  |  |  |  |  |  |

**SECTION A: FOR ALL AGE GROUPS.**

| **Not at all 🡪 Extremely** | | | | | | | | |
| --- | --- | --- | --- | --- | --- | --- | --- | --- |
| **Because of food allergy, my child…** | | **0** | **1** | **2** | **3** | **4** | **5** | **6** |
| 6 | Expresses physical distress |  |  |  |  |  |  |  |
| 7 | Expresses emotional distress |  |  |  |  |  |  |  |
| 8 | Has a lack of variety in his/her nutrition |  |  |  |  |  |  |  |

| **Not at all 🡪 Extremely** | | | | | | | | |
| --- | --- | --- | --- | --- | --- | --- | --- | --- |
| **Because of food allergy, my child has been affected negatively by…** | | **0** | **1** | **2** | **3** | **4** | **5** | **6** |
| 9 | The fact that he/she is receiving more attention than other children of his/her age |  |  |  |  |  |  |  |
| 10 | The fact that he/she has to grow up more quickly than other children of his/her age |  |  |  |  |  |  |  |
| 11 | The fact that his/her environment is more  restricted than other children of his/her  age |  |  |  |  |  |  |  |

| **Not at all 🡪 Extremely** | | | | | | | | |
| --- | --- | --- | --- | --- | --- | --- | --- | --- |
| **Because of food allergy, my child’s social**  **environment is restricted because of limitations on…** | | **0** | **1** | **2** | **3** | **4** | **5** | **6** |
| 12 | Restaurants we can safely go to as a family |  |  |  |  |  |  |  |
| 13 | Holiday destinations we can safely travel to  as a family |  |  |  |  |  |  |  |

**SECTION B: FOR CHILDREN AGED 4-12 YEARS**

| **Not at all 🡪 Extremely** | | | | | | | | |
| --- | --- | --- | --- | --- | --- | --- | --- | --- |
| **Because of food allergy, there are restriction on my child’s ability to participate in…** | | **0** | **1** | **2** | **3** | **4** | **5** | **6** |
| 14 | social activities in other people’s homes (sleepovers, parties, play time) |  |  |  |  |  |  |  |
| 15 | *preschool/school events which*  *include food (class parties, treats,*  *lunch)* |  |  |  |  |  |  |  |

| **Not at all 🡪 Extremely** | | | | | | | | |
| --- | --- | --- | --- | --- | --- | --- | --- | --- |
| **Because of food allergy, my child feels…** | | **0** | **1** | **2** | **3** | **4** | **5** | **6** |
| 16 | Worried when he/she goes to unknown places |  |  |  |  |  |  |  |
| 17 | Worried that he/she must always  be cautious with food |  |  |  |  |  |  |  |
| 18 | That he/she cannot take part in activities which include food |  |  |  |  |  |  |  |
| 19 | Upset that the family’s social activities have been limited due to the need to plan ahead |  |  |  |  |  |  |  |
| 20 | Concerned about accidentally eating a substance which he/she is allergic to |  |  |  |  |  |  |  |
| 21 | Worried when eating with unfamiliar adults/children |  |  |  |  |  |  |  |
| 22 | Anxious because of social restrictions |  |  |  |  |  |  |  |

| **Not at all 🡪 Extremely** | | | | | | | | |
| --- | --- | --- | --- | --- | --- | --- | --- | --- |
| **Because of food allergy, my child…** | | **0** | **1** | **2** | **3** | **4** | **5** | **6** |
| 23 | is more worried in general than other children of his/her age |  |  |  |  |  |  |  |
| 24 | is more careful in general than other  children of his/her age |  |  |  |  |  |  |  |
| 25 | Is not as confident as other children of his/her age in social situations |  |  |  |  |  |  |  |
| 26 | Wishes his/her food allergy would  disappear |  |  |  |  |  |  |  |

**SECTION C: FOR CHILDREN AGED 7-12 YEARS**

| **Not at all 🡪 Extremely** | | | | | | | | |
| --- | --- | --- | --- | --- | --- | --- | --- | --- |
| **Because of food allergy, my child feels…** | | **0** | **1** | **2** | **3** | **4** | **5** | **6** |
| 27 | Concerned about his/her future (opportunities, relationships) |  |  |  |  |  |  |  |
| 28 | That many people do not understand the serious nature of food allergy |  |  |  |  |  |  |  |
| 29 | Concerned about the insufficient labeling on food products |  |  |  |  |  |  |  |
| 30 | That food allergy limits his/her life in general |  |  |  |  |  |  |  |

**Thank you for completing this questionnaire. We would be grateful if you would now answer some questions about your child’s food allergy.**

**SECTION D: FOR ALL AGE GROUPS**

**Part 1:**

My child’s food allergy:

**Ε1**. What sex are you?

| MALE |  | FEMALE |  |
| --- | --- | --- | --- |

**Ε2**. What sex is your child?

| BOY |  | GIRL |  |
| --- | --- | --- | --- |

**Ε3**. What age is your child with food allergy? Years ____ Months ____

**Ε4**. What types of foods is your child allergic to? Tick all applicable options.

🞎 Peanuts 🞎 Nuts 🞎 Milk

🞎 Wheat 🞎 Soy 🞎 Egg

🞎 Shellfish 🞎 Fruits 🞎 Fish

🞎 Vegetables 🞎 Sesame 🞎 Other

Please define ‘other’:

**Ε5**. After ingesting which food did your child have his/her most severe reaction?

**Ε6**. Did your child have an anaphylactic reaction? Yes 🞎 No 🞎

**Ε7**. If ‘yes’, how recent was this reaction? Tick the applicable option.

🞎 Very recent

🞎 6 to 12 months ago

🞎 1 year ago, approximately

🞎 2 years ago, approximately

🞎 More than 2 years ago

**Ε8**.a. Has your child been treated with an anapen/adrenaline/epinephrine?

Yes 🞎 No 🞎

**Ε8**.b. What was the reason for the provision of an anapen/epipen?

1. Reassurance to you to your child
2. Concerns for you for your child

**Ε9**. Who diagnosed your child with food allergy?

| Family doctor |  |
| --- | --- |
| Allergist |  |
| Pediatrician |  |
| Dermatologist |  |
| Nutritionist |  |
| Other doctor |  |

**Ε10**. What symptoms does your child have in terms of food allergy? Tick all applicable options.

|  | Itching in the mouth |  |  | Wheeze |
| --- | --- | --- | --- | --- |
|  | Itching in the throat |  |  | Cough |
|  | Itching in the ears |  |  | Itching of the skin |
|  | Itching on the lips |  |  | Redness of the skin |
|  | Runny nose |  |  | Increased eczema |
|  | Stuffy nose |  |  | Urticaria |
|  | Sneezing |  |  | Skin swelling |
|  | Itchy eyes |  |  | Nausea |
|  | Tears |  |  | Abdominal cramps |
|  | Red eyes |  |  | Vomiting |
|  | Throat tightening |  |  | Diarrhea |
|  | Difficulty swallowing |  |  | Light-headedness |
|  | Hoarseness |  |  | Palpitations |
|  | Difficulty breathing |  |  | Inability to stand |
|  | Shortness of breath |  |  | Loss of consciousness |

**Ε11**. How often does your child meet another child with food allergy?

| Never |  |
| --- | --- |
| Rarely |  |
| Sometimes |  |
| Often |  |

**SECTION E: FOR ALL AGE GROUPS**

**Part 2:** Yours and your child’s worries concerning food safety.

| **0 = extremely unlikely**  **1 = very unlikely**  **2 = somewhat unlikely**  **3 = likely**  **4 = quite likely**  **5 = very likely**  **6 = extremely likely** |
| --- |

**Please answer the following questions with reference to the 6-point scale.**

**Ε1**. **What chance do YOU think your child has of..**

| **Question** | | **6-point scale** | | | | | | |
| --- | --- | --- | --- | --- | --- | --- | --- | --- |
|  |  | 0 | 1 | 2 | 3 | 4 | 5 | 6 |
| 1 | Accidentally swallowing a food that they are allergic to? |  |  |  |  |  |  |  |
| 2 | Having a severe reaction if the food is accidentally swallowed? |  |  |  |  |  |  |  |
| 3 | Dying from his/her food allergy after an ingestion in the future? |  |  |  |  |  |  |  |
| 4 | Effectively treating him/herself or receiving an effective treatment from others (including adrenalin) if he/she accidentally consumes food that he/she is allergic to? |  |  |  |  |  |  |  |

**Ε2**. What chance does your **CHILD** think he/she has of…

| **Question** | | **6-point scale** | | | | | | |
| --- | --- | --- | --- | --- | --- | --- | --- | --- |
|  |  | 0 | 1 | 2 | 3 | 4 | 5 | 6 |
| 1 | Accidentally swallowing a food that they are allergic to? |  |  |  |  |  |  |  |
| 2 | Having a severe reaction if the food is accidentally swallowed? |  |  |  |  |  |  |  |
| 3 | Dying from his/her food allergy after an ingestion in the future? |  |  |  |  |  |  |  |
| 4 | Effectively treating him/herself or receiving an effective treatment from others (including adrenalin) if he/she accidentally consumes food that he/she is allergic to? |  |  |  |  |  |  |  |

| **0-2** |  |
| --- | --- |
| **3-6** |  |
| **7-10** |  |
| **10+** |  |

**Ε3**. How many foods does your child have to avoid?

**SECTION Z: FOR ALL AGE GROUPS**

**Part 3:** Your concerns as a parent

**Ε1**. How would you describe

Α. **YOUR** general health?

| Excellent |  |
| --- | --- |
| Very Good |  |
| Good |  |
| Moderate |  |
| Not so Good |  |
| Poor |  |
| Very Poor |  |

Β. Your **CHILD’S** general health?

| Excellent |  |
| --- | --- |
| Very Good |  |
| Good |  |
| Moderate |  |
| Not so Good |  |
| Poor |  |
| Very Poor |  |

**Ε2**. Because of food allergy, how much does each of the following concern you?

Α. your child’s physical health?

| NOT AT ALL |  | A LITTLE BIT |  | SOME |  | QUITE A BIT |  | A LOT |  |
| --- | --- | --- | --- | --- | --- | --- | --- | --- | --- |

Β. your child’s emotional health?

| NOT AT ALL |  | A LITTLE BIT |  | SOME |  | QUITE A BIT |  | A LOT |  |
| --- | --- | --- | --- | --- | --- | --- | --- | --- | --- |

**Ε3**. How much stress does your child’s food allergy cause

Α. you?

| NOT AT ALL |  | A LITTLE BIT |  | SOME |  | QUITE A BIT |  | A LOT |  |
| --- | --- | --- | --- | --- | --- | --- | --- | --- | --- |

Β. your husband/partner?

| NOT AT ALL |  | A LITTLE BIT |  | SOME |  | QUITE A BIT |  | A LOT |  |
| --- | --- | --- | --- | --- | --- | --- | --- | --- | --- |

Γ. your family?

| NOT AT ALL |  | A LITTLE BIT |  | SOME |  | QUITE A BIT |  | A LOT |  |
| --- | --- | --- | --- | --- | --- | --- | --- | --- | --- |

**Ε4**. Has the food allergy limited the type of activities

Α. which can have as a family?

| NOT AT ALL |  | A LITTLE BIT |  | SOME |  | QUITE A BIT |  | A LOT |  |
| --- | --- | --- | --- | --- | --- | --- | --- | --- | --- |

Β. which your child can take part in?

| NOT AT ALL |  | A LITTLE BIT |  | SOME |  | QUITE A BIT |  | A LOT |  |
| --- | --- | --- | --- | --- | --- | --- | --- | --- | --- |

Thank you for taking the time to complete this questionnaire. Your participation is most appreciated.
